# Detection of herpes simplex virus in oral tongue squamous cell carcinoma

**DOI:** 10.3389/fphar.2023.1182152

**Published:** 2023-05-10

**Authors:** Tiina Koivikko, Priscila Campioni Rodrigues, Mari Vehviläinen, Petra Hyvönen, Elias Sundquist, Riikka K. Arffman, Ahmed Al-Samadi, Hanna Välimaa, Tuula Salo, Maija Risteli

**Affiliations:** ^1^ Research Unit of Population Health, Faculty of Medicine, University of Oulu, Oulu, Finland; ^2^ Medical Research Center Oulu, Oulu University Hospital, University of Oulu, Oulu, Finland; ^3^ Department of Oral and Maxillofacial Diseases, University of Helsinki, Helsinki, Finland; ^4^ Department of Health and Social Management, University of Eastern Finland, Kuopio, Finland; ^5^ Finnish Student Health Service, Helsinki, Finland; ^6^ Research Unit of Biomedicine, Faculty of Medicine, University of Oulu, Oulu, Finland; ^7^ Department of Virology, University of Helsinki, Helsinki, Finland; ^8^ HUSLAB, Department of Virology, Helsinki University Central Hospital, University of Helsinki, Helsinki, Finland; ^9^ HUSLAB, Department of Pathology, Helsinki University Central Hospital, University of Helsinki, Helsinki, Finland

**Keywords:** herpes simplex virus, oral tongue squamous cell carcinoma, viability, invasion, immunohistochemistry

## Abstract

**Introduction:** Oral tongue squamous cell carcinoma (OTSCC) is the most common cancer of the oral cavity. Contradictory results have been observed on the involvement of herpes simplex virus 1 (HSV-1) in oral squamous cell carcinomas. Here, we aimed to study the predominance of HSV-1 or HSV-2 in oral HSV infections and to investigate the presence of HSV-1 in OTSCC and its effect on carcinoma cell viability and invasion.

**Methods:** The distribution of HSV types one and two in diagnostic samples taken from suspected oral HSV infections was determined from the Helsinki University Hospital Laboratory database. We then analysed 67 OTSCC samples for HSV-1 infection using immunohistochemical staining. We further tested the effects of HSV-1 using six concentrations (0.00001–1.0 multiplicity of infection [MOI]) on viability and two concentrations (0.001 and 0.1 MOI) on invasion of highly invasive metastatic HSC-3 and less invasive primary SCC-25 OTSCC cell lines using MTT and Myogel-coated Transwell invasion assays.

**Results:** Altogether 321 oropharyngeal samples were diagnosed positive for HSV during the study period. HSV-1 was the predominant (97.8%) HSV type compared with HSV-2 (detected in 2.2% of samples). HSV-1 was also detected in 24% of the OTSCC samples and had no association with patient survival or recurrence. OTSCC cells were viable even after 6 days with low viral load (0.00001, 0.0001, 0.001 MOI) of HSV-1. In both cell lines, 0.001 MOI did not affect cell invasion. However, 0.1 MOI significantly reduced cell invasion in HSC-3 cells.

**Discussion:** HSV-1 infection is predominant compared with HSV-2 in the oral cavity. HSV-1 is detected in OTSCC samples without clinical significance, and OTSCC cell survival or invasion was not affected at low doses of HSV-1.

## 1 Introduction

Oral squamous cell carcinoma (OSCC), including oral tongue cancer (OTSCC), is the 18th most common malignancy worldwide (Sung et al., 2021), and the incidence of OTSCC has increased in recent decades (Tota et al., 2017). In a Finnish study, the 5-year recurrence-free survival of OTSCC was only approximately 65% (Mroueh et al., 2017). The most common risk factors for OTSCC are tobacco and alcohol consumption (Morse et al., 2007; Albuquerque et al., 2011; Tomo et al., 2020). However, several other factors, such as bacteria, viruses, diet, irradiation, and genetic mutations can promote OTSCC development (Petti, 2009; Hillbertz et al., 2012; Gupta and Johnson, 2014; Sand and Jalouli, 2014; Hettmann et al., 2015). Human papilloma virus (HPV) plays a pathogenic role in the head and neck SCC. However, its incidence in OSCC is less common when compared with oropharyngeal SCC (Sabatini and Chiocca, 2019). Additionally, the prevalence of HPV positive OSCC shows a significant heterogeneity in term of anatomical location and population (Katirachi et al., 2023).

Studies on the involvement of herpes simplex virus 1 (HSV-1) in the aetiology of OSCC and head and neck cancer (HNC) have yielded contradictory results (Parker et al., 2006; Sand and Jalouli, 2014). In some studies, HSV-1 antibody levels alone were associated with oral cancer (Shillitoe et al., 1984; Jain, 2016), and elevated levels predicted lower 5-year survival (Shillitoe et al., 1986). Furthermore, *in vitro* cell cultures, animal models, and patient studies suggest that HSV-1 may enhance cancer development by potentiating the tumorigenic effects of other risk factors, such as tobacco, alcohol and HPV (Hirsch et al., 1984a; Hirsch et al., 1984b; Stich et al., 1987; Cornella et al., 1989; Cox et al., 1993; Starr et al., 2001; Jalouli et al., 2012; Sand and Jalouli, 2014). On the other hand, other studies detected no association between OSCC and the presence of HSV-1 (Sand and Jalouli, 2014; Jalouli et al., 2015). One study even reported a lower risk of HNC in the presence of HSV-1 (Parker et al., 2006). Additionally, HSV-1 has been detected in ulcerative mucositis in HNC patients receiving radiotherapy (Nicolatou-Galitis et al., 2006).

In this study, we used immunohistochemistry to determine the distribution of HSV types in oral HSV infections and HSV-1 in paraffin-embedded specimens of OTSCC patients. Additionally, we studied the effects of HSV-1 infection on OTSCC cell viability and invasion.

## 2 Materials and methods

### 2.1 HSV detection in oral swab samples

We investigated the results of diagnostic oropharyngeal swab samples sent for analysis by type specific HSV rapid viral culture immunoperoxidase assay (Ziegler et al., 1988) at Helsinki University Hospital Laboratory during the period 1 January 2011 to 31 December 2014. The samples were acquired at tertiary, secondary, and primary care units from patients suspected of having HSV infection.

### 2.2 Immunohistochemistry

This retrospective study was approved by the Ethics Committee of the Northern Ostrobothnia Hospital District, Finland (49/2010, 56/2010) and the Finnish National Supervisory Authority for Welfare and Health (6865/05.01.00.06/2010). The formalin-fixed, paraffin-embedded blocks from patients with primary OTSCC treated between 1981 and 2005 were retrieved from the surgical pathology archives of the University Hospital of Oulu (*n* = 67). Detailed patient data are presented in Table 1.

**TABLE 1:** Baseline characteristics of patients with oral tongue squamous cell carcinoma.

Patient clinical data	
Age, years	
<60	22 (32.8)
≥60	45 (67.2)
Range	27–99
Mean	64
Median	66
Sex	
Male	34 (50.7)
Female	33 (49.3)
Tumour grade	
I, well	13 (19.4)
II, moderate	37 (55.2)
III, poor	14 (20.9)
Unknown	3 (4.5)
Tumour stage	
I	34 (50.7)
II	32 (47.8)
III	1 (1.5)
Neck lymph nodes	
Positive	36 (53.7)
Negative	25 (37.3)
Unknown	6 (9.0)
Adjuvant therapy	
No	33 (49.3)
Radiotherapy	24 (35.8)
Radiochemotherapy and chemotherapy	8 (11.9)
Unknown	2 (3.0)
Recurrence	
No	39 (58.2)
Yes	27 (40.3)
Unknown	1 (1.5)

All values are n (%) unless otherwise indicated.

Immunostaining was performed on 5-µm thick tissue sections using Herpes Simplex Virus I Rabbit Polyclonal Antibody (1:2000, Cell Marque). Briefly, after dewaxing and hydration in graded alcohol solutions, the antigens were retrieved by microwaving for 10 min in Tris-EDTA buffer pH 9.0 and cooled for 20 min at room temperature. Endogenous peroxidase was blocked using peroxidase-blocking reagent (S2023, DAKO) for 10 min. After washing with phosphate-buffered saline (PBS), the sections were treated with normal serum (Vector Laboratories, Burlingame, CA) in 2% bovine serum albumin/phosphate-buffered saline (BSA/PBS) for 30 min and then incubated with streptavidin-biotinylated horseradish peroxidase (StreptABComplex/HRP, Dako). Reactions were developed by incubating the sections with 3,3′-diaminobenzidine tetrahydrochloride (DAB, Vector) and counterstained with Mayer’s haematoxylin.

### 2.3 Cell culture

The following two human OTSCC cell lines were used: metastatic HSC-3 with high invasion potential (JCRB 0623; Osaka National Institute of Health Sciences) (Momose et al., 1989; Matsumoto et al., 1994) and primary SCC-25 with less invasion potential (ATCC, CRL 1628) (Ramos et al., 1997). Both cell lines are negative for HPV (Tugizov et al., 2005; Hirshoren et al., 2014). The cells were grown in 1:1 Dulbecco’s Modified Eagle Medium (DMEM)/Ham’s Nutrient Mixture F-12 (Gibco) supplemented with 10% heat-inactivated foetal bovine serum (FBS) (Gibco), 100 U/mL penicillin, 100 μg/mL streptomycin, 50 μg/mL ascorbic acid, 250 ng/mL amphotericin B, and 0.4 μg/mL hydrocortisone (all from Sigma-Aldrich). All cells were maintained at 37°C with 5% CO_2_. Cells were regularly tested for *mycoplasma* using EZ-PCR *Mycoplasma* test kit (Biological Industries, Beit-Haemek, Israel).

### 2.4 HSV-1 infection

Cells were infected with wild-type HSV-1 (strain 17+), of which the viral titre was verified by plaque titration on Vero cells with a standard protocol (Purves et al., 1987). For viability assays, 2000 HSC-3 cells and 3000 SCC-25 cells were seeded into 96-well plates as quadruplicates and incubated for 72 h at 37°C to achieve sufficient confluence. Cell number was counted before infection to determine the appropriate amount of virus to achieve predetermined multiplicity of infections (MOIs). The cells were infected at 0.00001, 0.0001, 0.001, 0.01, 0.1, and 1 MOI. Uninfected cells served as controls. The infections were performed by replacing the growth medium with 50 μL of medium containing the required amount of HSV-1 for each MOI. After 2 h of incubation at 37°C, the infection medium was removed and replaced with 100 μL of normal culture medium. In the 144-h cell viability assay, normal medium was again replaced for the cells 4 days after infection.

For the invasion assays, 70,000 HSC-3 and SCC-25 cells in 200 μL of serum-free medium containing 0.5% lactalbumin were infected in triplicates with 0.1 or 0.001 MOI. Cells were incubated on ice for 30 min with occasional stirring, after which infection medium was removed by centrifugation and replaced with fresh lactalbumin medium. Cells kept on ice without virus served as controls.

### 2.5 Cell viability assay

Cell viability was determined using MTT-based cell growth determination kit (Sigma) at 0, 24, 72, and 144 h. MTT solution was added to the cultures, which were then incubated at 37°C for 3 h. The culture medium was removed, and the formazan crystals were dissolved in isopropanol (MTT solvent). The plates were mixed for 10 min to enhance dissolution. Absorbance was measured at wavelength 550 nm with Victor 3 V multi-label reader (Perkin Elmer). Cell viability was represented as a percentage normalized with the uninfected cells at 0 h time point.

### 2.6 Transwell invasion assay

Transwell plates with 6.5-mm inserts, pore size 8 µm (Corning) were coated with 50 μL of Myogel extracellular matrix developed in our laboratory (2.4 mg/mL of protein, 0.2% agarose in serum-free medium) (Salo et al., 2015; Salo et al., 2018). The gels were left to set at room temperature for 30 min. Infected cells were added to Transwell inserts and 500 μL of normal culture medium was added to the bottom of the wells. Cells were incubated in 37°C until invasion was detected, which was 72 h for HSC-3 cells and 144 h for SCC-25 cells. Invasion was measured by toluidine blue staining (Salo et al., 2015). In brief, cells were fixed in 4% neutral-buffered formalin and stained with 1% toluidine blue in 1% borax solution. Excess dye was washed out and non-invading cells were gently removed from the upper part of the membrane with a cotton swab. The stained cells were then eluted in 1% SDS solution and absorbance was measured at 650 nm using a Victor2 Microplate Reader (Perkin Elmer Wallac).

### 2.7 Statistical analysis

*p*-values were calculated using One-Way Analysis of Variance (ANOVA) followed by Bonferroni correction. All analyses were performed in IBM SPSS Statistics 22 software (SPSS, Inc., Chicago, IL). *p* ≤ 0.05 was considered as statistically significant.

## 3 Results

### 3.1 HSV-1 is predominant in oral swabs; HSV-2 is rarely detected

**FIGURE 1:**
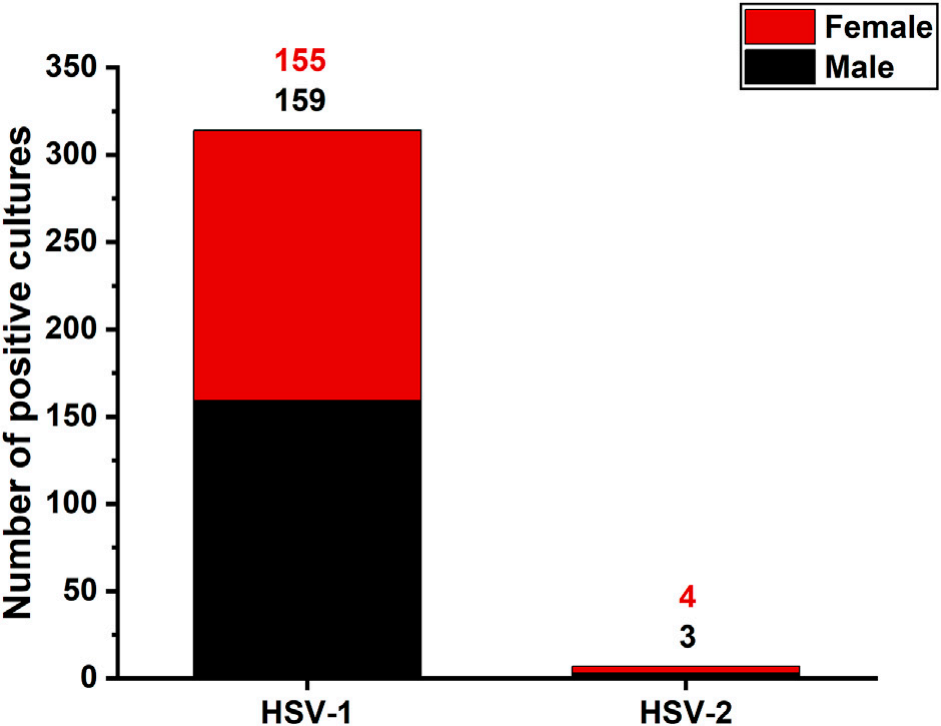
Detection of HSV-1 and HSV-2 in oropharyngeal swab samples from patients with suspected HSV infection. 321 swabs were cultured and the presence of HSV-1 and -2 was examined. HSV-1 was detected in 97.8% and HSV-2 in 2.8% of the studied swabs.

To investigate the epidemiologic distribution of HSV types in oropharyngeal infections, diagnostic laboratory data were reviewed to identify the HSV type used in vitro assays. Altogether 321 HSV positive oropharyngeal samples were diagnosed during the study period. Of these, HSV-1 was detected in 314 (97.8%) and HSV-2 in seven cases (2.2%) (Figure 1). The predominance of HSV-1 over HSV-2 was seen in both males and females (HSV-1/HSV-2: females 155/4, males 159/3).

### 3.2 HSV-1 infection detected in OTSCC samples

**FIGURE 2:**
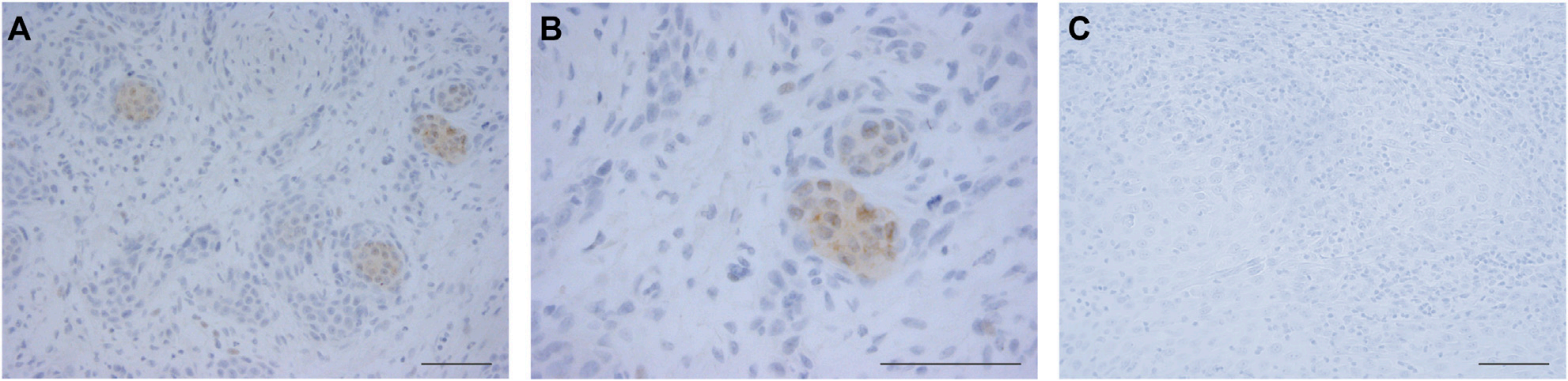
Expression of HSV-1 in OTSCC. OTSCC resection slides were stained for HSV-1. Positive OTSCC cells exhibited distinct nuclear and some cytoplasmic staining **(A,B)** compared with negative cells **(C)**. Pictures were taken at ×20 **(A)**, ×40 **(B)**, and ×20 **(C)** magnification. Scale bar 50 µm.

Sixty-seven OTSCC samples were immunostained for HSV-1. Positive staining was detected in 16 (24%) samples. The positive OTSCC cells exhibited distinct nuclear and some cytoplasmic staining (Figure 2). Kaplan-Meier estimates revealed no significant difference in patient survival or recurrence between HSV-1 positive and negative cases (data not shown).

### 3.3 Effects of HSV-1 infection on cell viability of OTSCC cell lines

**FIGURE 3:**
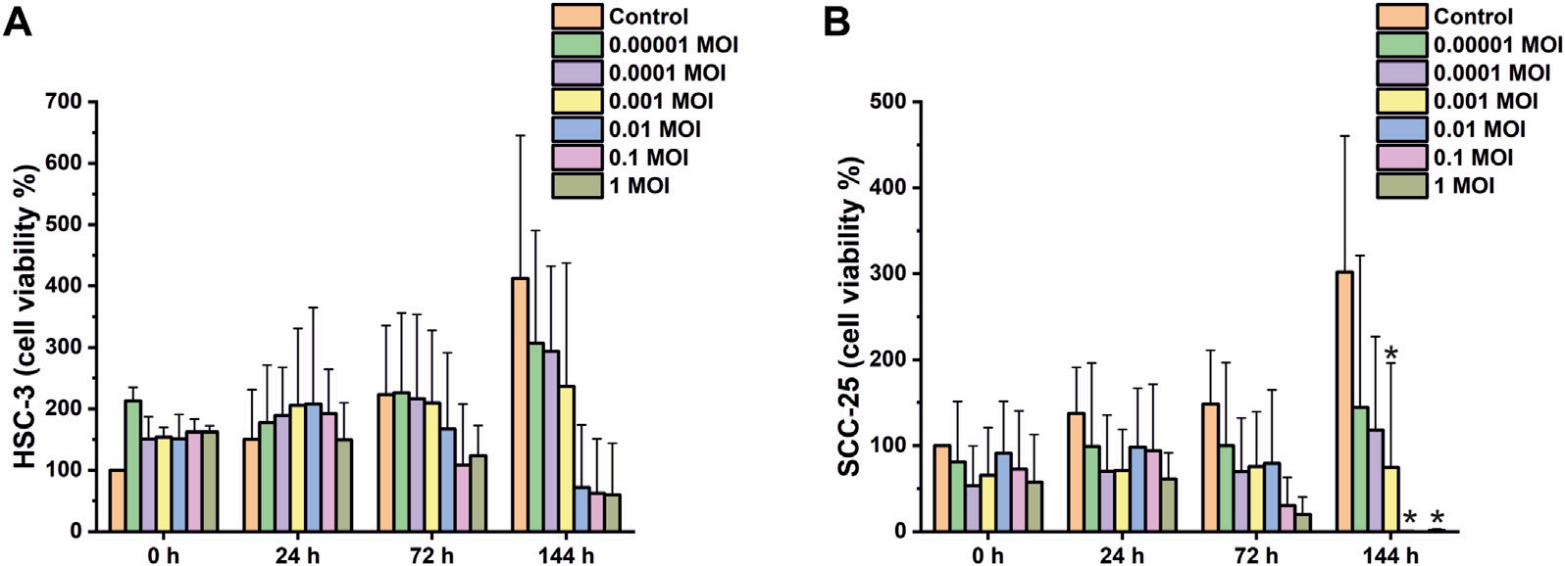
Viability assay of HSV-1 infected OTSCC cell lines. HSC-3 **(A)** and SCC-25 **(B)** cell viability shown as a percentage compared with uninfected at 0 h time point. Viability was measured using MTT-based cell growth determination kit. Cells were infected with indicated multiplicity of infection (MOI) of HSV-1. Viability ratings of >100% are due to cell proliferation during the experiment. The values represent the average ± SD of 2, 3 independent experiments. *p ≤ 0.05.

As HSV-1 was the predominant HSV type in oropharyngeal infections, we infected HSC-3 and SCC-25 OTSCC cell lines with six different viral loads and accessed cell viability with MTT assay at four time points. In the first 24 h, there was no difference in cell viability between control and infected cells (Figure 3). However, cell viability started to decrease after 72 h in cells infected with high dose of HSV-1 (0.01, 0.1, and 1 MOI; Figure 3). This effect was seen more clearly after 144 h and was observed in both HSC-3 and SCC-25 cells, however it was statistically significant only for SCC-25 cells.

### 3.4 Effects of HSV-1 infection on cell invasion of OTSCC cell line

**FIGURE 4:**
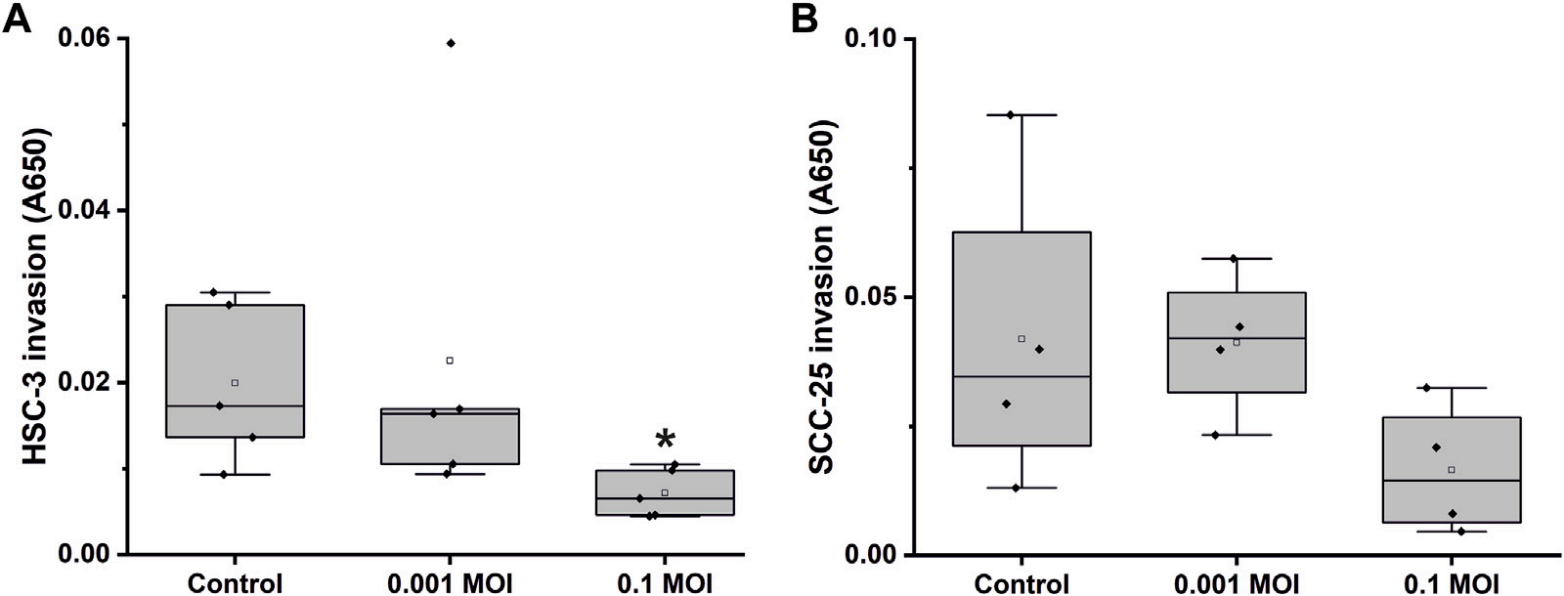
Transwell invasion assay of HSV-1 infected OTSCC cell lines. HSC-3 **(A)** and SCC-25 **(B)** cell invasion through Myogel-coated Transwell with 0.1 and 0.001 MOI of HSV-1. The invaded cells were stained with toluidine blue, stain was dissolved in 1% SDS solution, and absorbance at 650 nm was measured. The values represent the average ± SD of 4, 5 independent experiments. *p ≤ 0.05.

The invasive potential of HSV-1-infected OTSCC cell lines was analysed using Transwells coated with Myogel (Salo et al., 2015; Salo et al., 2018). Based on the viability assay, cells were infected with 0.1 and 0.001 MOI, representing virus loads that resulted in 35%–90% and 6%–50% viability of HSC-3 and SCC-25 cells at 72 h post-infection compared with uninfected cells, respectively (Figures 4A, B). Low dose of HSV-1 (0.001 MOI) did not have a significant effect on HSC-3 and SCC-25 cell invasion. However, higher dose of virus (0.1 MOI) reduced the invasion of both cell lines, which reached statistical significance only for the HSC-3 cell line.

## 4 Discussion

HSV-1 is suggested to be a co-carcinogen in OSCC, acting together with other carcinogens by potentiating their tumorigenic effects (Parker et al., 2006; Sand and Jalouli, 2014). To further investigate the effect of HSV in OTSCC, we first identified the HSV type most commonly detected in the oral cavity. In addition, we determined the presence of HSV-1 infection in OTSCC patient samples using immunohistochemistry and analysed the effect of HSV-1 infection on two OTSCC cell lines, the more aggressive HSC-3 and less aggressive SCC-25. Our data revealed that HSV-1 is predominant in oral-cavity HSV infections compared with HSV-2. We also showed that HSV-1 is detected in 24% of OTSCC cases and OTSCC cells can survive with low-dose HSV-1 infection.

While the role of HPV in oral cancer has been studied extensively (Simonato et al., 2016; Santos et al., 2021; Kirschnick et al., 2022), other viruses, such as HSV-1, have not been investigated in detail. Using immunostaining, we observed positive staining of HSV-1 in 24% of OTSCC samples. Our results were consistent with previous results indicating the presence of HSV in 15%–56% of oral cancer patients either by detecting HSV-1 IgG in the patient blood or by detecting viral DNA in brush biopsies or in formalin-fixed, paraffin-embedded oral carcinoma blocks by PCR (Jalouli et al., 2010; Jalouli et al., 2012; Jain, 2016). Our results showed that there was not a significant association between the presence of HSV-1 infection and patient survival. Our results were partially in contrast to a previous study that investigated the association between levels of IgM, IgG, and IgA antibodies to HSV-1 and oral cancer patient survival (Shillitoe et al., 1986). This study revealed an association between patient survival and IgM and IgG but not IgA levels. These contrasting results may be due to different methods used for evaluating the presence of HSV-1.

Cancer cell proliferation and invasion are important aspects in cancer progression and development. According to our results, HSV-1 was clinically the predominant HSV type in oropharyngeal infections. Therefore, we studied the effect of different doses of HSV-1 on cancer cell viability and invasion. As expected, high doses of HSV-1 (0.01, 0.1, and 1 MOI) seemed to be toxic to the cancer cells and decreased viability of the cancer cells, however this was not statistically significant for both cell lines and at all time points. Interestingly, OTSCC cells tolerated low doses of HSV-1 (0.0001, 0.0001, and 0.001 MOI) without a clear effect on viability. Our results are consistent with those of Spear et al. (2000), who showed a similar dose-dependent effect of HSV-1 on pancreatic carcinoma cells (Spear et al., 2000).

We selected two HSV-1 doses (0.001 and 0.1 MOI) to study the effect of HSV-1 on OTSCC cell invasion. The low dose of HSV-1 had no effect, but the higher dose reduced cell invasion of both cell lines, which is probably explained by the toxic effect of HSV-1 as seen in the viability assay. However, only the decreased invasion of HSC-3 cells reached statistical significance. Our results are consistent with a previous study reporting that HSV-1 infection at 0.5 MOI reduced melanoma cell spheroid number in 3D cultures (Valyi-Nagy et al., 2018). However, in hamster buccal pouches, a combination of HSV-1 and smokeless tobacco enhanced micro-invasive SCC (Stich et al., 1987). Additionally, HSV-1 antibody was associated with regional or distant spread of oropharyngeal SCC (Starr et al., 2001). Our results suggest that OTSCC with low-dose HSV-1 infection still have invasive ability.

This was a small study that aimed to reveal the presence of HSV-1 in OTSCC samples. Limitations of this study include the low number of samples used for immunohistochemical staining of HSV-1 and a missing control group for the oral swab samples.

In conclusion, our data showed that HSV-1 is far more common than HSV-2 in oral HSV infections, and HSV-1 can be detected in a subset of OTSCC patient samples. Additionally, HSV-1 infection was toxic to OTSCC cell lines in high doses, whereas survival was not affected with a low viral load. In accordance with the viability results, low dose did not affect OTSCC cell invasion, whereas higher doses reduced the invasive potential of the cancer cells.

## Data Availability

The original contributions presented in the study are included in the article/supplementary material, further inquiries can be directed to the corresponding author.
